# Surveillance of antimicrobial resistance in low- and middle-income countries: a scattered picture

**DOI:** 10.1186/s13756-021-00931-w

**Published:** 2021-03-31

**Authors:** Katia Iskandar, Laurent Molinier, Souheil Hallit, Massimo Sartelli, Timothy Craig Hardcastle, Mainul Haque, Halyna Lugova, Sameer Dhingra, Paras Sharma, Salequl Islam, Irfan Mohammed, Isa Naina Mohamed, Pierre Abi Hanna, Said El Hajj, Nurul Adilla Hayat Jamaluddin, Pascale Salameh, Christine Roques

**Affiliations:** 1 Department of Mathématiques Informatique et Télécommunications, Université Toulouse III, Paul Sabatier, INSERM, UMR 1027, 31000 Toulouse, France; 2 INSPECT-LB, Institut National de Santé Publique, d’Épidémiologie Clinique et de Toxicologie-Liban, Beirut, 6573-14 Lebanon; 3 Faculty of Pharmacy, Lebanese University, Mount Lebanon, Lebanon; 4 Faculté de Médecine, Equipe constitutive du CERPOP, UMR1295, unité mixte INSERM, Université Paul Sabatier Toulouse III, 31000 Toulouse, France; 5 Faculty of Medicine and Medical Sciences, Holy Spirit University of Kaslik (USEK), Jounieh, Lebanon; 6 Department of Surgery, University of Macerata, 62100 Macerata, Italy; 7 Department of Trauma Service, Inkosi Albert Luthuli Central Hospital, Durban, 4091 South Africa; 8 Department of Surgery, Nelson Mandela School of Clinical Medicine, University of KwaZulu-Natal, Congela, 4041 Durban South Africa; 9 Unit of Pharmacology, Faculty of Medicine and Defence Health, Universiti Pertahanan Nasional Malaysia (National Defence University of Malaysia), Kem Perdana Sungai Besi, 57000, Malaysia; 10 Faculty of Medicine and Defence Health, National Defence University of Malaysia, 57000 Kuala Lumpur, Malaysia; 11 Department of Pharmacy Practice, National Institute of Pharmaceutical Education and Research (NIPER) Hajipur, Bihar, India; 12 Department of Pharmacognosy, BVM College of Pharmacy, Gwalior, India; 13 Department of Microbiology, Jahangirnagar University, Savar, Dhaka-1342 Bangladesh; 14 Department of Restorative Dentistry, Federal University of Pelotas School of Dentistry, Pelotas, RS 96020-010 Brazil; 15 Pharmacoepidemiology and Drug Safety Unit, Pharmacology Department, Medical Faculty, Universiti Kebangsaan Malaysia, Jalan Yaacob Latif, Kuala Lumpur, Malaysia; 16 Department of Medicine, Lebanese University, Beirut, Lebanon; 17 Faculty of Medicine, University of Nicosia, Nicosia, Cyprus; 18 Department of Bactériologie-Hygiène, Centre Hospitalier Universitaire, Hôpital Purpan, 31330 Toulouse, France; 19 Department of Bioprocédés et Systèmes Microbiens, Laboratoire de Génie Chimique, Université Paul Sabatier Toulouse III, UMR 5503, 31330 Toulouse, France

**Keywords:** Surveillance, Antimicrobial resistance, Low- and Middle-Income countries

## Abstract

Data on comprehensive population-based surveillance of antimicrobial resistance is lacking. In low- and middle-income countries, the challenges are high due to weak laboratory capacity, poor health systems governance, lack of health information systems, and limited resources. Developing countries struggle with political and social dilemma, and bear a high health and economic burden of communicable diseases. Available data are fragmented and lack representativeness which limits their use to advice health policy makers and orientate the efficient allocation of funding and financial resources on programs to mitigate resistance. Low-quality data means soaring rates of antimicrobial resistance and the inability to track and map the spread of resistance, detect early outbreaks, and set national health policy to tackle resistance. Here, we review the barriers and limitations of conducting effective antimicrobial resistance surveillance, and we highlight multiple incremental approaches that may offer opportunities to strengthen population-based surveillance if tailored to the context of each country.

## Background

Low- and Middle-income countries bear the highest burdens of communicable diseases with potentially the least resources, and limited data on the epidemiology and burden of antimicrobial resistance (AMR) [1, 2]. Current information about the geographical distribution of resistance is limited, laboratory capacity may be underdeveloped, and the challenges of conducting comprehensive population-based surveillance are high [3–6]. The World Health Organization (2014) report on the global surveillance of AMR [2] highlighted the gaps in information on pathogens of major public health threats. The lack of high-quality data limits the ability to assess and monitor trends of resistance worldwide [2]. AMR surveillance systems are the core component of infectious disease management [6] and the foundation for a better understanding of the spread of antimicrobial resistance [7]. Data on the local, national, and international levels may serve to improve public health, inform health policies, trigger responses to health emergencies, provide early warnings of emerging threats, and identify long–term resistance trends [6]. High-quality surveillance data are key-prerequisites for the assessment of the economic burden of resistance [3, 4]. In Low- and Middle-income countries, AMR surveillance capability is variable [7]. Sub-Saharan Africa and South and Southeast Asia have the least developed coverage compared with high-income countries like the United States and the European countries [7]. In low-income settings, the challenges are enormous due to weak laboratory and communications infrastructures, limited resources, lack of trained and qualified staff, and multiple socioeconomic and behavioral drivers of resistance [8–11]. Data on AMR surveillance are fragmented and lack representativeness [12]. The sources of data are mainly tertiary hospitals, pharmaceutical companies, academia, the private sector, and supranational networks in the absence of health system governance and health system information [12–14]. Laboratory capacity building relies on funding that may be short-term limiting affecting the sustainability of the progress made. International initiatives aim to provide support, enhance cooperation, and support capacity building [12–14]. The Global Antimicrobial Resistance Surveillance System (GLASS) launched in 2015 [15] strived to support global action on AMR and strengthen evidence base surveillance. The scope of the study is AMR surveillance of bacteria in humans with no emphasis on malaria and tuberculosis. Through the article, Low- and Middle-Income countries (LMICs) refer to Low-Income Countries (LICs) and Middle-Income Countries (i.e. Lower Middle-Income Countries and Upper Middle-Income Countries).Here, we aim to show the limitations and challenges to implement the AMR surveillance system in Low- and Middle-income countries and assess the factors contributing to the scattered data on surveillance and the opportunities to conduct high quality comprehensive population-based surveillance.

## The particularities of low- and middle-income countries

The World Bank list of economies (June 2020) classifies 29 countries as low-income and 106 countries as middle-income (www.databank.worldbank.org › data › site-content › CLASS). According to the World Health Organization (2019) report [16], global spending on health is in transition. Estimates show an increase in health spending per year between 2000 and 2017 by 7.8% LICs and 6.3% in Middle-income countries. In 2017, the WHO estimated that the average health spending across Low-Income Countries (LICs) was US$ 41 per person that is 70 times lower than health expenditures per person in high-income countries [16]. Donor funding represents only 0.2% of health spending globally [16]. Poorly developed countries may rely heavily on funding to support disease control programs [13]. These funds are crucial as they have saved millions of lives [13, 16]. Governments in these countries may not consider health a priority [10]. Granting priority to health is a political choice [16]. Other issues are related to socio-economic and socio-behavioral challenges, and food safety, limited access to medications, inadequate or lack of health information systems, and reliance on funding [9–11]. In LICs, the spectrum of infectious disease differs compared with other regions worldwide [13]. Communicable diseases remain the leading cause of mortality and morbidity [17, 18]. Vector-borne diseases are on the rise and the ability to prevent and treat to combat large outbreaks in low-incomes settings remains challenging [17]. In the past 15 years, there has been dramatic progress in malaria, Human Immunodeficiency Virus/Acquired Immunodeficiency Syndrome (HIV/AIDS), Tuberculosis, neglected tropical diseases, and other communicable diseases [18, 19]. According to the WHO (2020) world health statistics [20], the African region still lags far behind the global average in the incidence of malaria, tuberculosis (TB), and HIV. The current ratio of maternal deaths is 525 maternal deaths per 100 000 live births in Africa that is seven times greater than the target set by the World Health Organization (WHO). Communicable diseases are still the leading cause of childhood death [20]. The WHO Sustainable Development Goals (2016–2030) offer hope in accelerating the process toward better health, better sanitation, clean water, and solutions to poverty issues [21]. In LMICs, health system governance is questionable and at different stages of development [22, 23]. Health system governance is a complex, multidisciplinary, and multidimensional process that requires coordination across multiple stakeholders and the partnership of the private sector and civil society [22, 23]. The World Health Organization defines the health systems as “A well-functioning health system working in harmony, is built on having trained and motivated health workers, a well-maintained infrastructure, and a reliable supply of medicines and technologies, backed by adequate funding, strong health plans and evidence-based policies” [23].

## Barriers for effective surveillance of AMR

### Weak laboratory infrastructure

In LMICs, the basic requirements for a functional laboratory infrastructure are not met [24–26]. These include issues with the quality of water, electricity supply, light sources, climate control and ventilation, biosafety requirements, limited internet coverage and connection speed, lack of soap/alcohol gel, dust, insufficient toilet facilities, inadequate construction that hinders deep cleaning [26]. Inadequate laboratory infrastructure can influence the quality and reliability of pathogen detection and antimicrobial susceptibility testing [27, 28].

### Limited staff capacity and training

Understaffing [24–26, 29], lack of dedicated staff, the limited number of trained clinical and laboratory personnel [13, 14, 26, 29] affect the adequacy of data management [30]. The number of microbiologists and healthcare professionals with expertise in the field is limited [5, 27]. Lack of established professional standards or profile of clinical microbiologists and post-graduates activates is an additional major problem [26]. The limited involvement of microbiologists in staff training and orientation, the lack of governance and leadership, out -of hours calls issues [31], and poor management are challenging barriers [5, 25, 26]. Leaders provide a focal point of activities, advocacy, and championing [5]. With the absence of guidance and logistical management related to stock and waste management and inventory control, the quality of activity and level of performance may be deeply affected [25, 26].

### Communication issues

Poor communication between laboratory staff and the medical team [32] is crucial. The lack of confidence in laboratory results [26] is related to delays in reporting results [13], lack of relevant reported information such as not providing minimum inhibitory concentration (MIC) [13] in addition to the frequent shortages of diagnostics and reagents. As a result, physicians may rely on clinical judgment and disregard test results [33] or may be reluctant to request a laboratory test [34–36]. Other causes may include the costs and inability of the patient to cover these expenses [13].

### Limited or lack of availability of consumables, diagnostics, and reagents

Omelet and colleagues (2018) [26] discussed the need for diagnostics and reagents adapted to developed countries [26]. High temperatures and humidity are harsh conditions that may affect the quality of diagnostics and reagents that require sustainable and secured cold chain storage. Environmental conditions may be harmful to electronic equipment and other consumables [37]. Shortage of items, lack or limited local manufacturing, substandard local quality [38], and the use of cheap, low-quality reagents, and diagnostics may affect the accuracy of laboratory results [25]. Supply chain issues that include strenuous regulations for air shipment delivery may lead to long delays and may challenge the need for secured cold chain storage. These problems in the supply chain may be particularly harmful to products sensitive to temperature and humidity [25, 26, 39]. In poor resources settings, there is a need for robust equipment that is easy to repair and requires maintenance at low cost, which consumes low energy such as electricity-free incubators [40] and autoclaves powered by solar energy [41, 42]. Due to the potentially low return of investment, there may be no special commercial interest in investing in the development of new diagnostics adapted for use in low-resource settings [5, 26]. More efforts are being invested in the field and testing innovative and low-cost diagnostic [26]. Omelet and colleagues (2018) [26] also highlighted the suggested use of the blood of a sheep breed adapted to tropical climates as an alternative for sheep blood, horse blood, and rabbit plasma [26, 43, 44].

### Questionable quality assurance

Laboratory Guidance for the selection, sampling, and transport of specimens is absent [26]. There is a limited quality assurance of the process, and no systematic monitoring of quality indicators [39]. Updating standard operating procedures and other documents are challenging [25] due to multiple languages and cultural barriers to the good understanding of such procedures [38]. External quality assurance schemes for all laboratories involved in AMR surveillance is also challenging [14]. Integration of bacteria standardized criteria by international guidelines such as the Clinical and Laboratory Standards Institute (CLSI) and the European Committee on Antimicrobial Susceptibility Testing (EUCAST) into automated antimicrobial susceptibility testing are lacking in LMICs [26, 45, 46]. Guidelines are mainly only available in English, and poorly updated and followed [39] or may be complicated for use by staff that lack expertise in microbiology [26].

### Relying heavily on funding

External funding to strengthen the laboratory capacity and implement AMR surveillance programs is granted by agencies like the Fleming fund, the WHO, and U.S Centers for Disease prevention and Control (CDC) [15]. Funding initiatives' primary goal is to improve AMR surveillance in LMICs. The United Kingdom (UK) Department of Health launched the Fleming Fund to support low-income countries in developing AMR surveillance systems [47]. The fund is aligned with the WHO’s Global AMR Surveillance System (GLASS) [whom glass] to support the Global Action Plan on AMR [1, 12]. To build capacity in LMICs, the Fleming fund awarded a total amount of 265 million pounds [15] to different countries. Bangladesh, India, Laos, Nepal, Pakistan, and Vietnam have been awarded Fleming Fund country grants to initiate or strengthen AMR surveillance activities [15]. The challenge resides in sustainability [1] of the progress when funding initiatives are short-term which highlights the need for internal funding and government engagement [14, 15]. Funding can support research and the creation of networks needed in specific circumstances to provide quality data such as the Institute for Health Metrics and Evaluation funded by a joint award from Wellcome, the UK Fleming Fund, and the Bill and Melinda Gates Foundation to gather, map and analyze disease and mortality attributable to drug-resistant infections. There have been multiple calls [48] for the development of a Global Antimicrobial Conservation Fund [49] to support Global Innovation Fund for non-commercial research to further support the provision of basic bacteriology services in low-resource settings [14, 49].

## The data challenge on surveillance of AMR in low- and middle-income countries

### The need for high-quality data

One of the five strategic goals of the WHO global action plan is to strengthen data on AMR through surveillance and research [3]. Data can serve to alert for emerging communicable diseases outbreaks, inform health policymakers, provide the evidence base for developing treatment guidelines and monitor the trends and spread of resistance [12, 13, 15, 30]. Data can inform the implementation of infection prevention and control programs such as antimicrobial stewardship programs [30, 50]. Aggregated multi-sectoral National Surveillance data on AMR help to track trends of resistance across sectors, benchmark data, and implement and update health policy to tackle AMR [50].

### Limitations of current data on AMR in low- and middle-income countries

Data management is one of the main challenges of AMR surveillance [51–53]. The lack of trained staff [26], limited experience and expertise in the field [26], lack of standardization of antimicrobials susceptibility testing [15], inappropriate sampling of the patient with suspected infection [51] and multiple sources of data from pharma, supranational networks, private laboratories, hospital laboratories, and national surveillance network lead to fragmented and scattered data [13]. The lack of standardization and heterogeneity of data leads to data that suffers from a lack of reliability and representativeness [15, 51–53]. Other factors are related to the limited use or lack of access to technology that facilitates data generation, analysis, sharing, and dissemination [15, 51–53].

### Sources of data

#### National AMR surveillance programs

In 2015, WHO launched the GLASS that establishes a standardized approach for the collection, analyses, and data sharing on AMR worldwide [12]. The GLASS project provides surveillance and laboratory guidance and offers the tools needed to support the AMR surveillance process [14]. The WHO requires to establish a national action plan as a first step in the process of implementing surveillance on AMR in humans [3, 7]. In 2018, 69 enrolled countries out of which 49 reported the rate of AMR [12]. The first report revealed a high level of resistance and showed the seriousness of the situation worldwide [54]. Each country is requested to establish its national organizational structure and determine the terms of reference [7]. The creation of a National coordinating center (NCC) reflects the government engagement to strengthen AMR surveillance and shows commitment to international society toward the global action plan to mitigate resistance [3, 7]. The function of the NCC is setting national strategic planning AMR surveillance and monitoring the implementation and the level of quality performance of the program at the national level [7]. The NCC also commissions a situational analysis of laboratory capacity building and assurance of the sustainability of AMR surveillance [7]. Seal and colleagues (2017) [7] considered that LICs are currently of limited capacity to implement the GLASS and proposed a roadmap for graduated alignment with the GLASS procedures. The guideline shows flexibility across settings based on the standard core protocols of the GLASS to help generate valid data and inform evidence-based interventions on regional, national, and international levels [7]. Despite the evidence base improvement detected in recent years, the laboratory capacity for AMR surveillance in LMICs is still thought of as limited and fragmented [3, 7]. The GLASS enables a standardized data collection and reporting of official national AMR data [12, 15] that secures data reliability and representativeness. The system allows the collaboration of the WHO with existing regional and national AMR surveillance systems through harmonized global standards to produce timely and comprehensive data. Three large regional surveillance networks implemented a report with the participation of LMICs, routine AMR surveillance data on the target pathogens as defined by the GLASS [12]. These networks include the European Antimicrobial Resistance Surveillance Network (EARS-Net) [55] and Central Asia and Eastern Europe (CAESAR) [56], Latin American (Red Latino americana de Vigilancia de la Resistencia a Los Antimicrobianos, ReLAVRA) [7, 57]. The EARS-Net [55] is a publicly funded network of EU countries national surveillance systems launched in 1998. The network collects data from member states on seven key pathogens only from invasive samples such as blood and cerebrospinal fluid. Data that originates from national AMR initiatives and/or a smaller subset of local laboratory networks and hospitals are uploaded to the central European Center for Disease prevention and control (ECDC) database, and annual reports are posted publicly on the website as open access, interactive data that allows creating maps and reports at the country level. Many Laboratories report data according to the Clinical and Laboratory Standards Institute (CLSI) or the European Committee of Antimicrobial Susceptibility Testing (EUCAST) clinical guidelines, although at present, many European countries are shifting to EUCAST clinical guidelines [55]. Participating European middle-income countries include Bulgaria as classified by the World Bank list of economies (June 2020). Central Asia and Eastern Europe (CAESAR) [56] aims to strengthen AMR surveillance in the WHO European region that are not part of the EARS-Net. The CAESAR is coordinated by the ECDC and is part of the GLASS project [56]. The following countries are enrolled in the CAESAR network database: Upper-Middle-Income: Albania, Armenia, Azerbaijan, Belarus, Bosnia, and Herzegovina, Georgia, Kazakhstan, Kosovo, the Russian Federation, Turkey, Turkmenistan, Serbia according to the World Bank list of economies (June 2020) and Lower-middle income: Kyrgyzstan republic, Moldova, Tajikistan, Ukraine, and Uzbekistan. Countries outside the European Union can become a member of the CAESAR network [56]. All enrolled laboratories are encouraged to use the EUCAST or the CLSI guidelines. Training mainly focused on the EUCAST methods, considered the most widely used in the European Region, and freely access methodology in various languages [56]. The PAHO/WHO launched the Latin American (Red Latinoamericana de Vigilancia de la Resistencia a Los Antimicrobianos, ReLAVRA) in 1996. ReLAVRA is one of the oldest and largest regional AMR surveillance networks worldwide [57]. The network of national reference laboratories reports the magnitude and trends of AMR in the Region, using routine data of microbiology laboratories. Data reported annually by each national reference laboratory (NRL), are collected from sentinel centers in different countries. The NRL external quality assurance program is coordinated by the National Administration of Health Laboratories and Institutes in Buenos Aires, Argentina. Enrolled upper-income countries include Argentina, Brazil, Colombia, Costa Rica, Cuba, Ecuador, Guatemala, Mexico, Paraguay, Peru, Dominican Republic, Venezuela according to the World Bank list of economies (June 2020) and Lower-middle income Bolivia, El Salvador, Honduras, Nicaragua according to the World Bank list of economies (June 2020). Guidelines are implemented for species identification and antimicrobial susceptibility testing (AST) such as CLSI enable data comparisons between countries.

#### Alternative sources for data generation in low- and middle-income countries

##### Pharma

Pharmaceutical companies establish global networks examining bacterial susceptibility mainly to evaluate drug performance [13, 14]. These networks generate high-quality data on bacterial susceptibility pre- and post-drug marketing to fulfill regulatory requirements. Over the years, a certain number of global networks were funded by pharmaceutical companies [58]. Examples of these networks “Assessing Worldwide Antimicrobial Resistance and Evaluation Program (AWARE) from Astra-Zeneca/IHMA in 2008-ongoing, “Community-Acquired Respiratory Tract Infection Pathogen Surveillance (CAR TIPS). From Bayer HealthCare Pharma 2009–2010, “The Comparative Activity of Carbapenem Testing (COMPACT)” from Janssen Asia Pacific, a division of Johnson & Johnson Pte Ltd 2008–2010 and the: International daptomycin surveillance programs” from JMI Laboratories, North Liberty, IA, USA 2011–2011 (12/21) [14]. The advantage is that isolates originated from global distribution, operating procedures for pathogens identification, and antimicrobial susceptibility testing is compatible with international standards. Testing done in an accredited laboratory enhances the quality of the generated data. One network “The Alexander project” has led to the discovery of new resistance mechanisms like macrolide resistance to *Haemophilus influenza* [59, 60]. The disadvantages are the potential lack of representativeness of what is called small markets, and limited support for building laboratory capacity in Low- and Middle-income countries or advise health policy and implementation of guidelines and results may not reflect the local burden of resistance [14].

##### Academia

An academic network may offer high-quality data and has many advantages compared with a pharma network [13, 14]. These networks target a clinical and policy topic and in-depth information for a specific population and have a higher impact on improving clinical and laboratory capacity in LMICs compared with pharma networks. This positive influence on participating laboratories was demonstrated by the ARMed study that led to an improvement in bacterial identification and antimicrobial susceptibility testing (AST) owing to the external quality assessment (EQA) program attached to the network [61]. Research-generated data may have potential limitations [13]. Data may be prone to different types of bias such as sampling bias, duplication bias and bias related to laboratory practice that may influence the validity or interpretation of surveillance data [13, 14, 62].

##### Private laboratories

Private laboratories may play a major role in the provision of high-quality data on AMR surveillance if they are accredited and operate according to quality standards provision of services compared with public laboratories [13]. In South Africa, 80% of the South African National Accreditation System (SANAS) belongs to the private sector. In India, the vast majority of laboratories accredited by the National Accreditation Board for Testing and Calibration Laboratories (NABL) are in the private sector [63]. The private laboratories are well-equipped and accredited by different national and international accreditation agencies [63]. Data generated from the private sector may serve as a proxy for mapping AMR [resistance map] as they provide extensive datasets for the studied populations [63]. Generated data may suffer from bias [13, 62] and lack of representativeness [64]. (Table 1).

**Table 1:** Selected countries AMR National Surveillance Programs

Countries	Bangladesh	Brazil	India	Lebanon	Malaysia	South Africa	Ukraine
Population^a^	163.05 million	211.05 million	1.37 billion	6.86 million	31.95 million	58.56 million	41.98 million
World bank country classification by income[66]	LMIC	UMIC	LMIC	UMIC	UMIC	UMIC	LMIC
GLASS-AMR^a^	Yes	Yes	Yes	Yes	Yes	yes	No
National action plan^a^	In place	In place	In place	In place	In place	In place	Developed^b^
National coordinating center^a^	Established	Established	Established	Established	Established	Established	
Number of enrolled national surveillance centers^a^	8	18	130	30	110	353	
Number of enrolled hospitals	0	11	65	30	42	350	
In patient/outpatient facilities	8 Inpatient/ Outpatient facilities	7 outpatient facilities	65 outpatient facilities	0	68 outpatient facilities	3 outpatient facilities	Tertiary care hospitals^b^
AST Standard^a^	CLSI	EUCAST/CLSI	CLSI	EUCAST/CLSI	EUCAST/CLSI	EUCAST/CLSI	EUCAST^b^
National Reference Laboratory^a^	Established	Established	Established	Established	Established	Established	In progress^b^
EQA	Provided	Provided	Not provided	Not reported	Provided	Provided	Provided^b^
Number of laboratories performing AST^a^	8	11	41	30	43	50	5^b^
AST provided for GLASS pathogens	Some pathogens		Some pathogens	Some pathogens	All pathogens	All pathogens	All pathogens for CAESAR^b^
EQA provided for bacterial identification^a^	Some labs	Not provided	All labs	Some labs	All labs	All labs	All labs^b^

AMR, Antimicrobial Resistance; CLSI, Clinical and Laboratory Standard Institute; EUCAST, European Committee on Antimicrobial Susceptibility Testing; GLASS, Global Antimicrobial Resistance Surveillance System; EQA, External Quality Assessment

^a^World Health Organization. Global antimicrobial resistance surveillance system (GLASS) report: early implementation 2020

^b^World Health Organization. Central Asian and Eastern European Surveillance of Antimicrobial Resistance: Annual report 2019

## A Snapshot of AMR Surveillance Programs and the Epidemiology of AMR in Low- and Middle-income countries

The countries included in the study provide a snapshot of data on AMR surveillance programs and the epidemiology of AMR in LMICs located in different continents (Table 1).

*Bangladesh* is a lower middle-income country (LMIC) with a population of 163.05 Million. Bangladesh is enrolled in the GLASS and has an established national coordinating center with an in-place national action plan [65, 66]. The reported data on the epidemiology of antibiotic resistance are from different sporadic studies. Bangladesh is a member country of the WHO-SEARO (The World Health Organization-South-East Asia Regional Office) and the Global Antibiotic Resistance Partnership (GARP) that aims to strengthen a National Strategy and Action Plan for AMR [67]. AMR prevalence in Bangladesh has been reported widely in animal husbandry, environment, and aquaculture [68–70]. The presence of various β-lactamase genes, extended-spectrum β-lactamase (ESBL), and different types of mobile colistin resistance (*mcr*) genes were found in Bangladeshi veterinary and environmental sources [71–74]. Antimicrobial-resistant bacteria and antibiotic resistance genes (ARGs) have been reported extensively in hospital and community-acquired infection [75–78], gastroenteritis [79, 80], urinary tract infection (UTI) [81, 82], respiratory tract infection [83], skin and tissue infection [75], blood-borne infections [84]. Most studied bacteria are *Escherichia coli* [84, 85], *Salmonella Typhi* [79], *Mycobacterium tuberculosis* [76, 77], *Vibrio cholerae *[86], *Proteus mirabilis, Streptococcus pneumoniae, Haemophilus influenza* [83, 87], *Acinetobacter baumanni *(*A. baumannii*) [88], *Pseudomonas aeruginosa *(*P. aeruginosa*) [78], *Staphylococcus aureus* [75], *Klebsiella pneumoniae *(*K. pneumoniae*) [82, 89], *Clostridium difficile* [80].

*Brazil* is an Upper Middle-Income country (UMIC) with a population of 211.05 Million. Brazil is enrolled in the GLASS and has an established national coordinating center with an in-place action plan [65]. The Ministry of Health in Brazil launched a pilot project of the AMR Surveillance Program in 2018 [65]. By 2022, the WHO GLASS report (2020) [65] expects an increase in the number of centers participating in the project to include at least 95 hospitals and seven outpatient clinics located in all 26 Brazilian states. Published studies in the field originate from both the academic research conducted in hospital setting and the Unified Health Care System of the nation. A 5 years AMR surveillance in Brazil reports that **a**mong more than 20,000 genes detected, the blaKPC gene isolated from *K. pneumoniae* predominates, followed by blaOXA-23 *Acinetobacter spp*. The blaOXA-48, known to be highly prevalent in European countries is rarely found in Brazil. According to GLASS 2020 early implementation results, the prevalence of *S. pneumoniae, S.aureus, Escherichia coli *(*E. coli*)*, Acinetobacter spp. and Salmonella spp. are between 70 to100% reported in blood culture according to AST results *[65, 90]*.* The rates of MRSA are high estimated up to 60% and are related to an endemic Brazilian clone. Resistance to vancomycin was first attributed to *Enterococcus faecalis*, which differs from the reported epidemiology of Enterococci in Europe and America [91]. *K. pneumoniae and Escherichia coli *(*E.coli*) isolates producing ESBL have a much higher prevalence (40%–50% and 10%–18%, respectively). Other Gram-negative bacteria (GNB) such as *carbapenem-resistant K. pneumoniae and carbapenem-resistant non-fermenting gram-negative bacilli *(*NFGNB*)* are* frequently reported in different studies conducted in various states in Brazil [89, 91–93] A multi-setting multistate survey showed that these GNB had high prevalence variability in the Intensive Care Units (ICU) across different settings [90, 91]. Among nonfermenters, carbapenem resistance is strongly related to SPM-1 *P.aeruginosa* and OXA-23 *A. baumannii* complex enzymes where a phenotype has also emerged in these isolates that are only susceptible to Colistin [91].

*India* is an LMIC with a population of 3.37 billion. India is enrolled in the GLASS and has a national coordinating center, and established a national action plan [65, 66]. In 2011, the Indian government initiated the ‘national policy for containment of antimicrobial resistance [94] and initiated various programs to track the AMR surveillance and promote rational use of antimicrobials [95, 96]. In 2013, the Indian Council of Medical Research (ICMR) established the Antimicrobial Resistance Surveillance and Research Network (AMRSN) [97] to promote antibiotic stewardship amongst clinicians and other healthcare workers. AMR is one of the top 10 national priorities in India. A National Action Plan on Antimicrobial Resistance (NAP-AMR) was launched for the years 2017 – 2021 [98]. AMR in India gained focus due to the controversial nomenclature of the New Delhi Metallo-beta-lactamase-1 (NDM-1) [99]. Research in the field originates from single-center[100–107]. In India, bacterial resistance to fluoroquinolones, cephalosporins, carbapenem, Beta-Lactam, and colistin is highly prevalent. The most commonly reported resistant strains are *E. coli *[103, 108, 109], *Salmonella *species (*spp.*) [110, 111], *Shigella spp.*[104, 112, 113], *Pseudomonas spp. *[102, 105, 108, 109], and *Acinetobacter spp. *[100, 106, 107]. A study conducted by the Government of India reported that more than 70% of isolates of *K. pneumonia* and almost half of all *P. aeruginosa* found resistant to fluoroquinolones and third-generation cephalosporins [110]. Resistance to carbapenems and faropenem is reported for different pathogens [114]^.^ In 2019, under the National Antimicrobial Resistance surveillance network (NARS-Net India) the National Centre for Disease Control received AMR Surveillance data from 21 sentinel surveillance laboratories in different States. *E. coli* was the most prevalent pathogen (33%) in inpatients and outpatient settings. The second commonly detected pathogens were *Klebsiella spp*. (22%) isolated in ICU where 5% of total blood isolates were resistant to colistin*. Klebsiella spp*. and *E. coli* showed high resistance to carbapenem and 3rd and 4th generation cephalosporins. *S. aureus* constitute (18%) of the total reported isolates with 66% resistance to cefoxitin and 1% resistance to linezolid. Other gram-positive bacteria include *Enterococcus spp*. with 5% resistance to linezolid and 13% resistance to vancomycin. Other isolated pathogens include *Pseudomonas spp*. (10%), *Enterococcus spp*. (9%), *Acinetobacter spp*. (8%) and *Salmonella Typhi and Paratyphi* (< 1%). High rates of resistant pathogens to most antibiotics in ICU settings is a major concern in India [115, 116].

*Lebanon* is a UMIC with a population of 6.86 million. Lebanon is enrolled in the GLASS and has an established national coordinating center and a national action plan on AMR [65, 66].Published data on the epidemiology of antibiotic resistance in Lebanon originates from scarce studies conducted in tertiary care settings [54, 58, 117–125]. Studies in the field were retrospective[54, 121, 123–125], mostly single-centered, and mainly examined the widespread of *Enterobacteriaceae* [117, 118, 120, 126, 127]. Results showed the high prevalence of OXA-48-mediated carbapenem-resistant *E. coli* and *K. pneumoniae* [58, 115, 128–131]. Pathogens of concern isolated in Intensive Care Units (ICU) are extensively-antibiotic resistant *A.baumanii*[121] OXA-48 [117] and OXA-23*-*mediated infections [122]^.^ Very few studies tackled the alarming spread of gram-positive resistant bacteria [124, 125], *Methicillin-Resistant Staphylococcus Aureus *(*MRSA*) [119, 125, 132], and *Streptococcus* *spp. *[133, 134]. Data on AMR lack completeness, timeliness, and representativeness.

*Malaysia* is a UMIC with a population of 31.95 million. Malaysia is enrolled in the GLASS and has an established national coordinating center with an in-place national action plan [65, 66].

In Malaysia, a study in the early 1990s has reported the resistance patterns of more than 36,000 microorganisms isolated in 6 general hospitals [135]. The National Antibiotic Resistance Surveillance (NSAR) program focus on national resistance trends in common pathogens such as *Staphylococcus aureus*, *Streptococcus pneumonia *(*S.pneumoniae*), *E.coli*, *K.pneumoniae*, *A.baumannii*, *P. aeruginosa, *and enterococci. Data from the NSAR program showed a high burden of extended-spectrum B-lactamase-(ESBL) producing Enterobacteriaceae and is of concern in the hospitals [135]. The high burden of Carbapenem-resistant Enterobacteriaceae (CRE) infections has increased from 28 reported cases in 2011 to more than 800 in 2016. The New Delhi metallo-B-lactamase-1 (NMD-1) gene first identified in carbapenem-*resistant K. pneumoniae* (CRKP) in 2010 showed increased spread from 0.3% in 2011 to 3.5% in 2018. Colistin-resistant bacteria showed a widespread trend among hospitalized patients in recent years [136, 137].

In 2019, NSAR [138] reported a reduction in resistance rates for most of the microorganisms and antimicrobials tested compared to 2018. The rates of resistance of *Staphylococcus aureus *(*S. aureus*), *S. pneumonia*, and *K. pneumoniae* are decreasing. For example, the MRSA rate decreased from 19.4% in 2018 to 15.0% in 2019. Resistance to vancomycin was lower in 2019 in both *Enterococcus faecalis, Enterococcus faecium, *and polymyxin B resistance remained at a low level. On the contrary, resistance rates have increased from the previous year in the majority of antimicrobials tested for *A.baumannii* and *P. aeruginosa*, including non-susceptibility to carbapenems (imipenem and meropenem). *A. baumannii* is isolated from patients in various departments [138]. Ampicillin resistance rate has remained as high as 71% for *Escherichia coli* isolated from urine and showed resistance rate to cefepime, cefuroxime, and ciprofloxacin [138, 139].

*South Africa* is a UMIC with a population of 58.56 million. South Africa is enrolled in the GLASS and has an established national coordinating center with an in-place national action plan [65, 66].

The national first report of the five years 2012–2017 [140] showed that the so-called ESKAPE pathogens (i.e. *Enterococcus faecium *(*E. faecium*)*, S. aureus, K. pneumoniae, A. baumannii, P. aeruginosa, and Enterobacter spp.*) had varied resistance rates and patterns across the country, comprising between 24 and 33% of all cultures. It was surprising that 75% of antimicrobial use in South Africa was in humans, rather than the higher rates of use in animals from other countries [140]. *K. pneumoniae* had 60–70% resistance patterns with *ESBL*, limiting the use of cephalosporins as first-line therapy, while there is an emerging carbapenem resistance, albeit lower. *E. coli* *ESBL showed 25*% resistance patterns and a worrying increased resistance to quinolones (especially in the Free State and KwaZulu-Natal provinces). *P. aeruginosa* and multi-drug resistant *A. baumanni* were only susceptible to colistin [140]. Interestingly a decline in *MRSA* was found [140]. There are four major burdens of disease (communicable, non-communicable, maternal and child-health-related and injury related). *K. pneumoniae* had 60–70% resistance patterns with ESBL, limiting the use of cephalosporins as first-line therapy, while there is an emerging carbapenem resistance, albeit lower. Interestingly a decline in *MRSA* was found [140]. These reports originate from hospital settings rather than community-related AMR, where much use of empiric antimicrobials are on a “best guess” basis by nurse-led clinics and general practitioners GP’s dictated often by government or other “essential medicines lists” and basic care protocols, such as the National Department of Health guidelines [140]. Recent literature reveals that similar AMR trends with variable regional patterns and across the private and public sectors [140]. Numerous publications are detailing the results of continuing surveillance programs in the teaching public sector and in the private sector to monitor AMR. Upon initiation of the system, a study examined the reliability and types of data quality at the NHLS using seven established facilities across SA. Results showed that the common organisms were *S. aureus, E. Coli, K. pneumoniae, and P. aeruginosa*, with AMR trending upwards over time from between 30–60% in the early period and up to 64–81% in the later period [141]. Interestingly the same group reported that the more recent surveillance showed for the 2014–2015 period that most commonly used antimicrobials had a Pseudomonas susceptibility of over 65% [142]. The challenge in the pediatric population is that blood cultures are seldom positively reported predominance of *Staphylococcus spp. and up* to 30% *ESBL-producing K. pneumoniae*[143]. While most of the surveillance is generated from teaching hospitals a study comparing district and tertiary facilities in KwaZulu-Natal province demonstrated AMR increasing from the district level to the tertiary facility, but with those referred upward having higher rates than those treated only in the district facility, but both facilities had increased rates for the longer-stay patients (> 48 h) [144, 145].

Other studies have monitored the prevalence of various mutations and resistance patterns in parts of SA and show that there is a difference in the patterns between the private and public sectors, specifically *E. coli* (19% in the public sector versus 36% in the private sector), *A. baumannii* (14% public versus 4% private), *P. aeruginosa* (7% public versus 11% private) and S. aureus (27% public versus 17% private), however concerning was the rapidly decreasing carbapenem susceptibility among Enterobacteriaceae [146]. Susceptibility data indicated changing patterns in both sectors towards an increase in non-susceptibility to carbapenems in *K. pneumoniae*. Similar results were found in a longer period using data from KwaZulu-Natal [147], who also documented amikacin sensitivity in many *A. baumanii* specimens examined, with only 5.4% resistant to this medication [148]. These latter findings correlate with the experience of the trauma ICU at the quaternary KwaZulu-Natal facility, where selective treatment of *A. baumanii* is practiced [149, 150]. Worryingly recent research findings suggest that the acquisition of blaNDM-1-bearing plasmid structure, horizontal transfer, and clonal dissemination facilitate the spread of carbapenemases in SA and this bodes poorly for the availability of suitable antimicrobials to treat *K. pneumoniae* soon as this defeats the carbapenem group of antimicrobials [151, 152].

*Ukraine* is an LMIC with a population of 41.98 million. Ukraine is enrolled in the CAESAR network [65, 66]. In 2019, Ukraine set the National Action Plan of AMR to improve regulations and strengthen surveillance of the spread of resistance [153]. Published studies in the field originate from large tertiary care centers [153–160]. The Surveys of Antibiotic Resistance (SOAR) (2016–2017) conducted to determine the antibiotic susceptibility of *S. pneumonia,* and *H. influenzae isolates from community-acquired respiratory tract infections showed high susceptibility to tested antibiotics and an increase *in antibiotic resistance to trimethoprim/sulfamethoxazole and macrolides among *S. pneumoniae*[161–164]. The survey data considered *the EUCAST/CLSI and pharmacokinetics and pharmacodynamics breakpoints165*. Another study conducted in hospital settings to examine the pathogens associated with surgical site infections showed that most isolated pathogens were Gram-positive bacteria where staphylococci showed the highest resistance to Gentamycin and ceftibuten. Besides, data showed the prevalence of 48.1% *MRSA and 36.6% Methicillin-resistant S. epidermidis* (*MRSE*) while *vancomycin-resistant* S. aureus (VRSA) and vancomycin-resistant S. epidermidis (VRSE) ranged from 9.3% and 18.3%154. Another study conducted in acute care settings of isolates demonstrated the high prevalence of resistant pathogens causing healthcare-associated infections (HAI). Results showed that a total of 14.2% of enterococci were resistant to vancomycin and 28.2% of isolated *S. aureus* were methicillin-resistant, and 35.1% of Enterobacteriaceae were resistant to third-generation cephalosporins among which the attributable highest rates of resistance to the *K. pneumoniae* (53.8%) and *E. coli* (32.1%)[159]. A retrospective analysis of strains isolated from the patients with respiratory tract infections found increasing AMR resistance of *P. aeruginosa* and *A. baumanii *[165]. A ten-year surveillance study of pathogens implicated in urinary tract infections revealed a significant increase in the proportions of multidrug-resistant bacteria and fluoroquinolone-resistant *E. coli* [166]. The recent studies examined the prevalence of MRSA and *Methicillin-resistant Staphylococcus epidermidis* (MRSE) and *ESBL* production among Enterobacteriaceae in postpartum mastitis [167] and postpartum endometritis [168]. To date, molecular epidemiological studies are limited in Ukraine [169, 170].

## Discussion

A comprehensive AMR surveillance data is compiled from multiple sources worldwide across different sectors including the human health, animal health, and the agricultural sector interface, in addition to local and regional sources including primary and tertiary hospitals, laboratories, clinics, primary clinical settings [171–173], pharmaceutical companies, supranational and international networks, and academia [12–14]. Generated data are shared with national and potentially international surveillance systems [171–173]. The main barriers are the lack of standardization of data management, the lack of quality assessment and accreditation of the sources of data, and the lack of quality checks on data collection, analysis, reporting, and sharing [5, 12–14, 25, 26]. As a result, data is subject to bias such as sampling bias and duplication, which may have limited representativeness [12].

In LMICs, the challenges are high [5, 6, 12–14, 25, 26]. Inadequate health systems governance, absence of health system information, lack of laboratory capacity and infrastructure[50], limited government engagement, loose rules and regulations, lack of resources, and limited staff with adequate experience, expertise, training, and experience in the field are additional limitations [5, 6, 12–14, 25, 26]. The scarcity of financial resources and reliance on funding to strengthen laboratory capacity is an additional problem because these investments are usually short-term [13, 14]. This issue can influence the sustainability of the progress made if the government did not implement internal funding and health prioritization plan to strengthen the health systems tackle resistance [13, 14]. Other challenges include supply shortages, supply chain issues, counterfeit products, environmental challenges, diverse socio-economic drivers of resistance, and absence of leadership [26]. For all these factors, the epidemiology of AMR in LMICs is a scattered puzzle picture that needs to be rebuilt piece by piece to generate high-quality data. Based on our expertise in the field and literature review, we propose to highlight the pillar that can bring together the scattered pieces to complete the picture (Table 2).

**Table 2:** The contributory factors to the scattered picture on AMR surveillance in LMICs

Contributory factors	Potential issues	Proposed interventions
Weak Laboratory infrastructure	Inadequate construction including[24–28]: Quality of water Electricity supply Light sources Climate control and ventilation Biosafety requirements Limited internet coverage and connection speed Lack of infection prevention and control products Insufficient toilet facilities	National action plan Gap analysis Sentinel sites Cross borders and International cooperation Laboratory Accreditation Periodic audits for quality assurance and control Strict national rules and regulations Standard operating procedures Funding
Limited staff capacity and training	Understaffing[24–26, 29] Lack of dedicated staff Lack or Limited number of trained clinical and laboratory personnel[13, 14, 24–26] Lack or limited number of microbiologists and healthcare professionals with expertise in the field Lack or limited number of staff trained in data management process	Government plan for strengthening health workforces Educational grants for continuous education and training Educational grants for post-graduate education and specialization National coordinating committee Interventions of specialized scientific societies in the field Enhancement of postgraduates programs Continuous trainings on-site and off-site, l Establishing mandatory number of continuous credit per year for license eligibility Training on standard operating procedure, data management and on Antimicrobial susceptibility testing standards
Communication issues	Lack of trust between prescribers and laboratories due to[13, 15, 26, 32, 34, 36]: Lack of standardized Antimicrobial susceptibility testing Lack of expertise or unqualified staff Lack of trust in diagnostic products and equipment’s Laboratory items and diagnostics shortages	Role of the national coordinating committee in improving communication Accreditation of laboratories t improve quality Quality control for diagnostics manufacturing Standardization Rules and regulations to solve the supply chain issues Standard operating procedures National awareness and education programs
Limited or lack of Availability of Consumables, Diagnostics, and Reagents	Environmental factors like high temperature and humidity may affect the transport, storage and quality of the supplies Questionable supply chains Low quality of locally manufactured diagnostics High maintenance cost of equipment’s High energy consumption for equipment’s High waste generation Supply shortages[15, 25, 26, 37, 38, 41, 42]	Quality control of local manufacturer diagnostics Standardization f quality requirements for good manufacturing practices Government role in preventing supply shortages and supervision of the supply chain Periodic audit Diagnostics and reagents adapted to the harsh environment Funding local manufacturing Examining alternatives to save energy and providing low cost services
Relying heavily on Funding	Grants and funds may be short-term[15] Lack of national budget for internal funding	Government intervention for internal funding Role of National coordinating committee National action plan Re-structuring funds to invest in educational grants for better sustainability
Poor data management	Fragmented, low quality data that lack of reliability and representativeness[13–15, 26]	Standardization Aligning systems Training on data collection Management and analysis Data sharing International support through training, and use of new technologies Grants

### Government engagement, commitment and leadership

The mainstay to strengthen the laboratory capacity is government engagement. Health is a political decision [16]. The WHO [174] constitution principles state that “Governments have a responsibility for the health of their peoples which can be fulfilled only by the provision of adequate health and social measures” and “The enjoyment of the highest attainable standard of health is one of the fundamental rights of every human being without distinction of race, religion, political belief, economic or social condition”. When the government prioritizes health and the community wellbeing and aims for building a better country for its future generation, one of the main goals is to tackle AMR. Evidence-based data show the detrimental impact on health and economy in LMICs that bear the highest burden of communicable diseases [1]. The government plays a major role in health systems governance and collaborates with multiple national and international stakeholders to set health policy rules and regulations like antibiotic use and the problem of counterfeit products in addition to secure a reliable supply of other medicines and technologies depending on the country needs [22, 23]. The government must set a national multi-sectoral health plan of the human, animal, and agriculture interfaces and establish evidence-based policies to prevent and treat diseases [172, 173]. The government has the responsibility to conduct gap analysis in the field for a better understanding of the country AMR context, drivers, challenges, and trends and prioritization of expenditures on programs to tackle resistance and for building health systems information and laboratory capacity [22, 23]. The government assesses the health workforce for health coverage [175] and to fulfill the engagement toward achieving pre-set sustainable goals [176]. Other important decisions include setting a surveillance focal point and a national coordinating center (NCC) that enhances the national to international collaboration [177]. The NCC can enhance communication and collaboration between the prescribers and the local and national laboratories generated data by highlighting and addressing the gaps and contributory factors to the lack of trust [177]. Reaching these goals means less empirical treatment, less use of broad-spectrum antibiotics, and better disease management, better patient health, and antibiotic use [176]. To enhance AMR awareness, government collaboration with different national and international stakeholders can plan education and training on antimicrobial susceptibility testing standards and data management across different disciplines [177].

### Re-structuring external funding for better progress sustainability

International funding agencies, donors, and philanthropic organizations can invest in the manufacturing of adapted or alternative supplies and scholarship grants for post-graduate education, continuous education and training, and health systems research [14, 26]. Investing in education and high-quality research will bridge the lack of sustainability gap.

International initiatives, academia, Pharma, and specialized societies can also contribute to improving human resources knowledge through education and training [15, 26]. Investing in continuous staff training and support can also be undertaken internally by the clinical microbiologist and other healthcare professionals with high experience and expertise in the field [6, 26]. The assessment of the feasibility and the affordability of the surveillance program is the first step before implementation. A six-month surveillance program in Ghana demonstrated the feasibility of the project [27]. Concerning the affordability, establishing a reliable estimate of the costs of implementing a comprehensive global surveillance system in humans, animals, and in the environment is very challenging. Kenya, one of the countries participating in the East Africa Public Health Laboratory Network (EAPHLN), is constructing a national AMR surveillance network at an estimated cost of US $160,000 [7]. This cost can inform baseline estimates for the global allocated budget for implementing such a program in other similar countries [2]. The World Bank has recommended the estimated US $ 9 billion per year for the containment of AMR, about half of which is for building core veterinary and human public–health capacity in LMICs with the collaboration of different health policymakers among which the WHO [2]. This figure may sound very expensive but looking at the priceless long-term benefits and the countless cost of inaction will shift the paradigm from cost to a high level of return on investment [178]. Another challenge is enhancing laboratory infrastructure that can be tackled by proceeding incrementally by priority according to the settings as part of the national action plan [15, 25, 26]. The major problem remains in the funding to strengthen laboratory capacity that may be short term, and mandates finding other sources of investments by prioritizing health [13, 15].

### Laboratory accreditation and standardization of operating procedures

The key to secure high-quality performance is the national and international accreditation of laboratories that should undertake a periodic EQA embedded as a policy in the national action and national health plan [14, 15, 25, 26]. Laboratories should operate according to national rules and regulations but to standard operating procedures that should be revised and updated regularly. Staff must be aware, educated, and offered periodic training to make sure the same process is applied in each shift to secure laboratory results [13–15, 26, 177]. Securing the supply chain and investing in adapted equipment, diagnostics, and reagents adapted to the environmental, logistic, financial challenges of LMICs is crucial [26]. Locally manufactured diagnostics must comply with standardized norms that secure the quality and efficacy of these products [25, 26]. Seal and colleagues (2017) [177] proposed investing in the implementation of sentinel surveillance sites starting with one site that may have its laboratory or access to laboratory and should operate according to core capacity and aspire for high standards. This site can support the development of another site [177]. The authors propose a roadmap for implementation and consider that in case there is no capacity for coordinating an AMR laboratory, a cross border collaboration can also be applicable.

### Standardization of the data management process

Appropriate data management is an additional issue [13]. Better data means informed evidence base decision-making leading to enhanced accountability and efficient allocation of resources to fight AMR [4, 131]. Factors that can influence the quality of data are the methodology of data collection and the adequacy of data interpretation and analysis [55]. Other challenges include the use of unified internationally accepted techniques and clinical breakpoints guidelines, considered crucial for the interpretation of antibiotic susceptibility testing (AST) results [179]. The limitations of conducting comprehensive population surveillance on AMR are high in LMICs [5, 13, 15, 26]. Some countries may report data from national surveillance systems with broad population coverage, and others may report data from a subset of local laboratories, clinics, and healthcare settings focusing on one area and limiting the representativeness of data on the national level [55]. As the validity of surveillance systems relies on the comparability of participating laboratories [180], each may have different trends of AMR surveillance and different level of capacity for identifying the microorganisms and may show differences in the applied methodology and quality assurance limiting benchmarking [55, 181, 182]. Other inconsistencies across participating laboratories include differences in sampling, the use of different clinical case definitions, and the heterogeneous healthcare utilization [181]. Standardization of data management is the most challenging task if health information technology and adequate training are lacking.

### Capitalizing on new technologies

Bioinformatics and genomics offer a promising shift in paradigm [183–186]. With the massive progress made in the field of Bioinformatics in recent years, especially in the field of molecular biology, new research is conducted on how to implement this technology in antimicrobial resistance surveillance. Whole Genome Sequencing (WGS) techniques are getting increasingly affordable in many laboratories throughout the world [187]. The increase in usage of WGS of organisms could eventually replace standard techniques such as performing in vitro bacterial culture and exposing pathogens to different concentrations of antibiotics. The rationale behind the constant search for newer techniques lies in the remarkable increase of multidrug-resistant organisms that requires rapid actions in the treatment and administration of the accurate drug [188]. Standard bacterial culture and antimicrobial testing can take several days while WGS takes only 24 h and could be used for rapid diagnosis and in urgent cases [180].

However, the comparison of big data and analysis of genomes requires a professional approach that can link all this information. Effectively, Bioinformatics integrates knowledge from molecular biology, infectious disease specialty, and epidemiology to predict AMR. Multiple prediction categories like the Binary classification and Multiclass classification are used [189–191]. The three main approaches in bioinformatics are the identification of known antimicrobial resistance genes from WGS data, tracking gene expression in response to antimicrobials, and agnostic gene identification from pangenome analysis. The first approach is the one that could be applied clinically to detect AMR in pathogens. For example, the Typewriter method uses a database called BLASTn to compare genome sequence with WGS data of 24 ARG and their mutations in *S. aureus* species [189, 192].

This approach requires adequate resource allocation, interdisciplinary effort, funding, and teamwork to provide early diagnosis and increase the quality of care in the era of drug resistance. Low and middle-income countries such as in the Middle Eastern Arabian Peninsula are lagging in the research in WGS based research and might consider implementing these genomics techniques to gather AMR data for global surveillance [191]. Finally, new initiatives for tracking resistance are emerging internationally, especially in outbreaks and public health investigations such as in Europe [193].

*Artificial intelligence* (AI) is a new paradigm to combat AMR[194]. AI models have a significant performance in improving infectious diseases worldwide [195], controlling the spread of resistance[196, 197].The AMR surveillance methods rely on the identification and characterization of the epidemiology of antimicrobial-resistant pathogens[198].The phenotypic tests and the whole-genome sequencing (WGS) are two methods used to diagnose the antimicrobial susceptibility testing (AST)[198, 199]. The phenotypic tests examine the bacterial response in the presence of an antimicrobial agent, and the WGS characterizes the genome of the bacterial isolates[198, 199]. Both types of testing have limitations. Antimicrobial susceptibility testing (AST) is determined using the classic phenotypic methods by reference broth microdilution or a surrogate test like disc diffusion[198]. The resulting minimum inhibitory concentration (MIC) is interpreted against internationally standardized breakpoints like the CLSI and EUCAST to determine whether the pathogen is susceptible or resistant[198]. The phenotypic tests have multiple limitations not addressed by the WGS method that may include potential ambiguity in interpretation, methodological problems for some drugs like colistin, and bacterial species like slow-growing and fastidious bacteria, the disc diffusion tests may be affected by physical and chemical factors like the incubation period and the content of the growth media[198]. Besides, standard approaches may not be suitable for anaerobic or rare bacterial species[198].The WGS data are digital, and the tests are computer-based for better standardization and reproducibility, providing greater interlaboratory comparability. WGS method can be used for AMR surveillance on a national and local basis to compare several genomes from different sites, analyze local or regional transmission chains or networks, and trace sources of AMR infection outbreaks[198]. On a global level, AI technologies can serve multiple functions like monitoring pathogen populations, detecting high-risk AMR clones and groups at risk of infections, correlating virulence factors with patients outcomes, and assessing the impact of these interventions[198]. AMR surveillance using the WGS method helps to identify pathways of AMR evolution and molecular mechanisms underlying resistance. The WGS method requires phylogenetic analysis, variant analysis tools, strain typing, in combination with epidemiologic and clinical metadata, data on antibiotic use in addition to reference databases for genomic and AST data. This method necessitates large and high-dimensional datasets for efficient data extraction[194, 200], in addition to substantial and sustained financial investment, an established infrastructure, and previous professional experience in WGS analysis[200]. In Low- and Middle-income countries, these conditions are not available[198, 200]. The international standard operating procedure, regulatory guidelines, and quality assurance for using the WGS method for AMR surveillance are not currently available[198]. AI offers to improve the limitations of the previous technologies'[201–203].The success of AI depends on the comprehensiveness of data and the quality of databases containing the big clinical data[194, 204]. The challenges of obtaining evidence-based AMR surveillance remain the lack of standardized data and periodic updates[194, 205–207]. AI techniques used different methods to improve AST that include the combination of flow cytometer-assisted antimicrobial susceptibility test (FAST) and machine learning techniques[203] and IR-spectrometer method that combines infrared (IR) spectroscopy with the artificial neural network[208]. For WGS-AST, the Support Vector Machine (SVM) and the Set Covering Machine (SCM) models are used to learn and predict AMR phenotypes[179, 209].The SCM model allows genotype-to-phenotype predictions [192]. The SVM model uses the number of co-occurring k-mers between the genome of the isolates and the reference genes to learn and predict the phenotypes of the bacteria to a specific antimicrobial[194]. SVM is a binary classification model[179] that is considered a promising tool for AMR surveillance [194].

## Current gaps in research and Future directions

AMR surveillance from a “One Health” perspective is needed in LMICs for data comparability cross-borders and for mapping and tracking the spread of resistance. Aggregated data can advise the estimation of the economic burden of AMR from societal and ecosystem perspectives and examining the cost-effectiveness of the current infection control and prevention program. Prioritizing health expenditures is crucial in LICs. Another topic of interest is working on sampling the environmental resistome in these regions to explore the resistance determinants and understand the niches that contributed or will potentially contribute to infections with antibiotic resistance microorganisms.

## Conclusion

A significant progress is achieved toward standardization of a population-based AMR surveillance in LMICs. The mainstay remains that “One size-fits-all” global action cannot be applicable, and an efficient action plan starts by an understanding of the particularities of each country and by aligning regional, national and international efforts. The government commitment to health as a national priority is the key to strengthen regulations, follow-up the implementation of a national action plan and control anthropogenic activities on antibiotic use in different sectors.

## Data Availability

Not applicable.

## Abbreviations

AIDS: Acquired Immunodeficiency Syndrome
AMR: Antimicrobial Resistance
AMRSN: Antimicrobial Resistance Surveillance and Research Network
ARGs: Antibiotic resistant genes
AST: Antimicrobial susceptibility testing
AWARE: Assessing Worldwide Antimicrobial Resistance and Evaluation
CAESAR: Central Asia and Eastern Europe
CAR TIPS: Community-Acquired Respiratory Tract Infection Pathogen Surveillance
CDC: U.S Centers for Disease prevention and Control (CDC)
CLSI: Clinical and Laboratory Standards Institute
COMPACT: Comparative Activity of Carbapenem Testing
CRE: Carbapenem-resistant Enterobacteriaceae
CRKP: Carbapenem-resistant Klebsiella pneumonia
EAPHLN: East Africa Public Health Laboratory Network
EARS-Net: European Antimicrobial Resistance Surveillance Network
EQA: External Quality Assessment
ESBL: Extended-spectrum β-lactamase
EUCAST: European Committee on Antimicrobial Susceptibility Testing
GARP: Global Antibiotic Resistance Partnership
GLASS: Global Antimicrobial Resistance Surveillance System
GNB: Gram-negative bacilli
HIV: Human Immunodeficiency Virus
ICMR: Indian Council of Medical Research
LICs: Low-Income Countries
LMICs: Lower and Mmiddle-income countries
mcr: Mobile colistin resistance
MIC: Minimum inhibitory concentration
MRSA: Methicillin-resistant *Staphylococcus aureus*
MRSE: Methicillin-resistant *Staphylococcus epidermidis*
NABL: National Accreditation Board for Testing and Calibration Laboratories
NAP-AMR: National Action Plan on Antimicrobial Resistance
NCC: National coordinating center
NDM-1: New Delhi Metallo-beta-lactamase-1
NRL: National reference laboratory
NSAR: National Antibiotic Resistance Surveillance
ReLAVRA: Red Latino americana de Vigilancia de la Resistencia a Los Antimicrobianos
SANAS: South African National Accreditation System
SOAR: Surveys of Antibiotic Resistance
TB: Tuberculosis
UK: United Kingdom
UMIC: Upper Middle-Income country
WGS: Whole Genome Sequencing
WHO: World Health Organization
WHO-SEARO: World Health Organization-South-East Asia Regional Office
